# Chronic Treatment with a Water-Soluble Extract from the Culture Medium of *Ganoderma lucidum* Mycelia Prevents Apoptosis and Necroptosis in Hypoxia/Ischemia-Induced Injury of Type 2 Diabetic Mouse Brain

**DOI:** 10.1155/2015/865986

**Published:** 2015-04-06

**Authors:** Meiyan Xuan, Mari Okazaki, Naohiro Iwata, Satoshi Asano, Shinya Kamiuchi, Hirokazu Matsuzaki, Takeshi Sakamoto, Yoshiyuki Miyano, Hiroshi Iizuka, Yasuhide Hibino

**Affiliations:** ^1^Laboratory of Immunobiochemistry, Faculty of Pharmaceutical Sciences, Josai University, Saitama 350-0295, Japan; ^2^Laboratory of Organic and Medicinal Chemistry, Faculty of Pharmaceutical Sciences, Josai University, Saitama 350-0295, Japan; ^3^Laboratory of Pharmacology, Faculty of Pharmaceutical Sciences, Josai University, Saitama 350-0295, Japan; ^4^Department of Pharmaceutical Sciences, International University of Health and Welfare, Tochigi 324-8501, Japan; ^5^Noda Shokukin-kogyo Co. Ltd., 295 Nanakohdai Noda, Chiba 278-0051, Japan

## Abstract

Type 2 diabetes mellitus has been known to increase systemic oxidative stress by chronic hyperglycemia and visceral obesity and aggravate cerebral ischemic injury. On the basis of our previous study regarding a water-soluble extract from the culture medium of *Ganoderma lucidum* mycelia (designed as MAK), which exerts antioxidative and neuroprotective effects, the present study was conducted to evaluate the preventive effects of MAK on apoptosis and necroptosis (a programmed necrosis) induced by hypoxia/ischemia (H/I) in type 2 diabetic KKAy mice. H/I was induced by a combination of unilateral common carotid artery ligation with hypoxia (8% O_2_ for 20 min) and subsequent reoxygenation. Pretreatment with MAK (1 g/kg, p.o.) for a week significantly reduced H/I-induced neurological deficits and brain infarction volume assessed at 24 h of reoxygenation. Histochemical analysis showed that MAK significantly suppressed superoxide production, neuronal cell death, and vacuolation in the ischemic penumbra, which was accompanied by a decrease in the numbers of TUNEL- or cleaved caspase-3-positive cells. Furthermore, MAK decreased the expression of receptor-interacting protein kinase 3 mRNA and protein, a key molecule for necroptosis. These results suggest that MAK confers resistance to apoptotic and necroptotic cell death and relieves H/I-induced cerebral ischemic injury in type 2 diabetic mice.

## 1. Introduction

Type 2 diabetes, which is characterized by hyperglycemia associated with insulin resistance, is the most common metabolic disease in nearly all countries and is increasing explosively in developing countries [1]. The metabolic disorder of diabetes leads to characteristic complications contributing to the development of micro- and macrovascular atherosclerosis [2]. Patients with diabetes not only are predisposed to stroke but also often suffer exacerbated poststroke damages [3–5]. In accordance with these clinical observations, basic studies have also demonstrated that the diabetic state aggravates cerebral ischemic injury in both type 1 [6–8] and type 2 diabetic animal models [9, 10]. The major causes exacerbating postischemic cerebral damage with diabetes are considered to be elevated levels of oxidative stress and inflammatory cytokines. Sustained hyperglycemia has been suggested to produce excessive intracellular reactive oxygen species (ROS) and enhance systemic oxidative stress, which accelerates apoptotic and proinflammatory processes in the brain tissue [11]. Furthermore, visceral obesity associated with type 2 diabetes also has been reported to induce production of inflammatory cytokines and augment oxidative stress via dysregulation of adipose tissue function [12, 13].

Oxidative stress and subsequent inflammation are also involved in both the pathogenesis and development of cerebral ischemic injury [14, 15]. After ischemia, different types of cell death, that is, necrosis and apoptosis, occur depending on the severity of hypoxia, ATP depletion, and the vulnerability of cells in the ischemic region [16]. Reperfusion after a long period of vessel occlusion triggers an explosive generation of ROS such as superoxide radical (O_2_^−^), hydroxyl radical, hydrogen peroxide, and nitrogen species [17]. Necrosis, an uncontrollable cell death, rapidly occurs mainly in the ischemic core region, and subsequently, apoptosis is induced in the ischemic penumbral region by oxidative damage to lipids, proteins, and DNA [15]. On the basis of these findings, neuroprotective therapy with free radical scavengers to remove ROS and rescue the cells in the ischemic penumbra is developing for the treatment of acute ischemic stroke coupled with thrombolytic therapy [18].

In nearly a decade, necroptosis, as a novel type of programmed and controllable cell death with pathogenomic features similar to those of necrosis, has proved to contribute to various tissue pathological injuries, including neuronal cell death in cerebral ischemia [19, 20], neurodegeneration [21], and viral infections in the CNS [22], drawing interest toward necroptosis as a new target for therapy of these diseases. Necroptosis has been revealed to be triggered by the tumor necrosis factor (TNF) superfamily, such as TNF, Fas ligand, and TNF-related apoptosis inducing factor [23, 24]. TNF activates TNF receptor 1, recruiting complex I comprising receptor-interacting protein kinase 1 (RIP1) and other factors [25]. RIP1 deubiquitination dissociates the proteins of complex I from the receptor and induces transit of death-inducing signaling complex (DISC, complex II) consisting of RIP1, RIP3, caspase-8, and Fas-associated death domain protein to the cytosol. Normally, caspase-8 triggers apoptosis by activating the classical caspase cascade; whereas, under the inhibition of caspase-8, RIP1 and RIP3 form the necrosome by cross phosphorylation, leading to necroptosis [25, 26]. Thus, the execution of necroptosis requires the activation of RIP3 by a caspase-independent mechanism [27]. Recently, it has been shown that experimental ischemia increases the expression of RIP3 both in* in vitro* primary cultures of hippocampal neurons and* in vivo* rat hippocampal CA1, suggesting a contribution of RIP3 upregulation to the necroptotic process [28, 29]. There are a number of studies regarding the therapeutic approach for necroptotic cell death in cerebral ischemia [20, 21]; however, the explorations of these effective compounds have only recently begun.

Many oriental herbal/traditional plant medicines with antioxidative activities have been demonstrated to prevent and cure lifestyle-related diseases, including type 2 diabetes and its complications, in humans and animals.* Ganoderma lucidum* (*G. lucidum*) is a very popular medicinal fungus used in traditional Chinese medicine; it has an extensive variety of pharmacological activities responsible for its health benefits such as antioxidant, anticancer, anti-inflammatory, and immunomodulatory activities [31]. The functional food derived from this fungus, a water-soluble extract from the culture medium of* G. lucidum* mycelia (MAK), is a freeze-dried powder of a hot-water extract prepared from a solid culture medium composed of bagasse and defatted rice bran overgrown with* G. lucidum* mycelia; this food has a considerable background of 18 years of contributing to the health of consumers. Our previous reports revealed that chronic oral pretreatment with MAK relieves an exacerbation of cerebral injury induced by middle cerebral artery occlusion (MCAO) and reperfusion in streptozotocin- (STZ-) induced type 1 diabetic rats [32, 33]. MAK suppressed the augmentation of oxidative stress and expression of a number of proinflammatory genes or proteins, such as TNF-*α*, interleukin- (IL-) 1*β*, cyclooxygenase- (COX-) 2, and myeloperoxidase (MPO) in the STZ-induced diabetic rat brain, and decreased apoptotic cell death induced by MCAO/reperfusion in the ischemic penumbra [33]. These results imply that the cerebroprotective effects of MAK are mainly attributed to its antioxidant and anti-inflammatory properties. However, its efficacy in the ischemic injury of the type 2 diabetic animal model associated with obesity and insulin resistance has not been estimated. Therefore, in the present study, we investigated the protective effects of chronic oral pretreatment with MAK on cerebral ischemic injury induced by hypoxia/ischemia (H/I) in genetically obese diabetic KKAy mice, a model of human metabolic disorders. Our findings indicate that chronic pretreatment with MAK prevents H/I-induced enhancement of the expression of RIP3 and decreases necroptotic cell death as well as apoptosis in KKAy mice.

## 2. Materials and Methods

### 2.1. Preparation of MAK

MAK was prepared by Noda Shokukin-kogyo Co., Ltd. (Chiba, Japan), as previously described [34].* G. lucidum* mycelia were cultured in a solid culture medium composed of bagasse and defatted rice bran for approximately 3-4 months just before the formation of the fruit body. The whole culture medium with a dense growth of the mycelia was extracted with hot water, subjected to filter sterilization and thereafter freeze-dried to obtain MAK.

### 2.2. Animals and Treatments

Male KK-A^y^/TaJcl mice (7 week old) were purchased from CLEA Japan, Inc. (Tokyo, Japan). Each mouse was separately caged under the temperature-controlled environment (23 ± 0.5°C) and relative humidity (55 ± 10%) with a 12/12 h light-dark cycle. The mice were given a standard rodent chow (CE-2, CLEA Japan, Inc.) and water* ad libitum*. After acclimating for 1 week, the mice were divided into two groups; the MAK group, which was orally administered MAK (0.3 or 1 g/kg) dissolved in distilled water, and the control group, which was administered only distilled water. A volume of 0.05 mL/10 g body weight of the solutions was given via a stomach tube once daily during the next 7 days. All experiments were performed in compliance with the Guiding Principles for the Care and Use of Laboratory Animals approved by the Japanese Pharmacological Society, and the guidelines were approved by the Ethics Committee on Animal Care and Animal Experimentation at the Josai University (number H23033). The number of animals used was kept to the minimum necessary for meaningful interpretation of the data. Animal discomfort was also minimized.

### 2.3. Cerebral H/I

At 9 weeks of age, KKAy mice were subjected to unilateral cerebral H/I (8% O_2_, balance N_2_, 20 min, and 36.0°C), as previously described [35]. Mice were anesthetized with 2% halothane and 30% O_2_ balance air; a small incision was made in the neck, and the right carotid artery was isolated and double-ligated with a 4-0 surgical thread. After the incision was sutured, mice were allowed to recover mobility for access to food and water for 3 h. Then, the mice were exposed to systemic hypoxia for 20 min in a cylindrical glass chamber (10 cm diameter × 15.5 cm high) filled with 8% O_2_/balance N_2_, which was submerged in a 36.0°C water bath. The core body temperature of mice was maintained at 37.5–37.7°C. After the hypoxic exposure, mice were abruptly reoxygenated in the room air and returned to their cages with free access to food and water. The sham operation was given with the same manipulation without ligation of the right carotid artery and H/I to the control-sham and MAK-sham groups. All the mice were euthanized at 24 h after reoxygenation and the brain and plasma samples were collected.

### 2.4. Rotarod Performance Test

Impairments in motor coordination and balance in the mice were assessed by the accelerating rotarod test [36]. Mice were placed on a 3 cm diameter cylinder of the rotarod apparatus (MK-630B; Muromachi Kikai Co. Ltd., Tokyo, Japan) and accelerated from 4 to 40 rpm in 5 min. Trials began by placing the mouse on the rod and starting the rotation. Each trial ended when the mouse fell off the rod and the latency was recorded. Mice were given five trials 2 days before H/I. Then the rotarod performance was tested for three trials 2 h before and 24 h after H/I.

### 2.5. Neurological Score

Neurological evaluation was accomplished by a blinded observer before H/I and 24 h after H/I using the 18-point neurological scoring system [30]. This neurological score tests for spontaneous activity, motor impairments, and sensorial function. Severe impairments were graded 0 or 1 and no observed deficits were graded 3 (Table 1).

### 2.6. Infarct Size Assessment [8, 32, 33, 37]

At 24 h of reoxygenation, the mice were deeply anesthetized with halothane and decapitated. The brain was removed and cut into 2 mm coronal sections in a precooled mouse brain matrix. Slices were stained with 2% 2,3,5-triphenyltetrazolium chloride (TTC; Sigma-Aldrich, St. Louis, MO, USA) at 37°C for 20 min and then fixed with 10% formaldehyde (Wako Pure Chemical Industries, Ltd., Osaka, Japan). Infarct areas were determined using an image analysis system (Scion Image 1.62) and were integrated to obtain the infarct volumes per brain. Corrected infarct volume (%) = [left hemisphere volume − (right hemisphere volume − the infarct volume)]/left hemisphere volume × 100.

### 2.7. Hematoxylin & Eosin (H&E) Staining [37]

Mice were anesthetized and transcardially perfused with phosphate buffer (pH 7.4) followed by 4% paraformaldehyde (PFA). Mice brains were then removed and postfixed for 24 h in PFA at 4°C. Isolated brains were embedded in paraffin wax, sectioned (3-4 *μ*m thick), and stained with H&E, sequentially. The sections were evaluated by scanning the ischemic penumbral areas of the somatosensory cortex and hippocampus containing the CA1 regions under low-power magnification (40x) and then confirmed under higher power magnification (100x, 200x, and 400x). All histopathological scoring and evaluation were performed by an investigator without knowledge of the treatment. Images were obtained under a microscope (Olympus BX51; Olympus, Tokyo, Japan) equipped with a DP72 digital camera (Olympus).

### 2.8. Systemic Oxidative Stress

The plasma levels of hydroperoxide as a marker of total oxidative stress were measured by an active oxygen-free radical autoanalyzer (Free Radical Elective Evaluator: F.R.E.E.) using the diacron-reactive oxygen metabolites (d-ROMs) test kit as previously reported [32–34, 38]. The results of the d-ROMs test were expressed in arbitrary units called “Carratelli units” (*CARR U*), where 1* CARR U* corresponds to 0.08 mg/100 mL H_2_O_2_. F.R.E.E. and the kits were purchased from Diacron International s.r.l. (Grosseto, Italy).

### 2.9. O_2_^−^ Production

Intracellular O_2_^−^ generation in the ischemic penumbral areas of the somatosensory cortex and the hippocampal CA1 was detected by dihydroethidium (DHE) staining [8, 39]. Coronal brain sections (8 *μ*m thick) were incubated with DHE (10 *μ*mol/L, Sigma-Aldrich) in 10 mM phosphate-buffered saline (pH 7.4) for 30 min at 37°C. After washing and mounting the sections in slide glasses, three microscopic fields at the cortex and the CA1 of each hemisphere were captured using a laser scanning confocal microscope (FluoView FV1000, Olympus Co. Ltd., Tokyo, Japan). The fluorescence intensity of oxidized DHE in each field was quantified using an Imaging software (FV10-ASW 1.7, Olympus).

### 2.10. TUNEL Staining

Apoptosis in the brain tissues was evaluated by the TUNEL method with the “Apoptosis* in situ* Detection Kit Wako” (Wako Laboratories) [8, 33], which is based on the TUNEL (terminal deoxynucleotidyl transferase- (TdT-) mediated dUTP nick end labeling) procedure, that is, the addition of fluorescein-dUTP to 3′-terminals of apoptotically fragmented DNA with TdT, followed by immunochemical detection using anti-fluorescein antibody conjugated with horseradish peroxidase and 3-3′-diaminobenzidine tetrachloride as a substrate. The coronal brain slices (8 *μ*m thick) were lightly counterstained with hematoxylin and observed under a microscope (BX51W1, Olympus). Quantification of TUNEL-positive cells was achieved by cell counting in the hippocampal CA1 and the penumbral cortex region affected by H/I [33, 40]. Three randomly chosen visual fields were counted in each region by an investigator without the knowledge of the experimental conditions. The percentage of apoptotic cells was calculated by the apoptotic index as the number of positive-staining nuclei by the total number of nuclei.

### 2.11. Immunohistochemistry [8, 33]

The brain was perfused with cold saline and fixed with phosphate buffer (pH 7.4) containing 4% formaldehyde. Coronal brain sections (8 *μ*m thick) were incubated with blocking buffer (4% w/v, Block Ace; Dainippon Sumitomo Pharma Co. Ltd., Osaka, Japan) for 2 h. The sections were incubated with polyclonal rabbit anti-cleaved caspase-3 or RIP3 antibody (1 : 100, Abcam, Cambridge, UK) in 1% w/v Block Ace overnight at 4°C. After washing with PBS, these were correspondingly incubated with Cy3-conjugated secondary antibody (1 : 100, Chemicon, Temecula, CA, USA) for 2 h at room temperature. Finally, nuclei were stained with 4′,6-diamidino-2-phenylindole (DAPI; Invitrogen, Carlsbad, CA, USA) for 15 min at room temperature, washed, and mounted using a mounting medium with 80% glycerol. Immunofluorescence was visualized using the fluorescence microscopy as described above. The percentage of cleaved caspase-3-positive cells was calculated as the number of the positive-staining nuclei to the total nuclei [33, 40]. Regarding RIP3, because a lower level of expression of this protein was observed in every cell in the hippocampus, the cells having an enhanced expression of RIP3 under a laser beam at constant intensity were determined as RIP3-positive. All histopathological scoring and evaluation was performed by an investigator without knowledge of the treatment.

### 2.12. Real-Time PCR Analysis

The temporal gene expression patterns of apoptotic markers (Bcl-2 and Bax) and necroptosis-related factors (RIP1, RIP3 and TNF-*α*) were evaluated by real-time RT-PCR analysis [8, 33, 41]. The total RNA from the ischemic penumbral cortex region at 6 or 24 h of reoxygenation was extracted with the RNeasy Micro Kit (Qiagen, Hilden, Germany), according to the manufacturer's instructions. Total RNA (0.5 *μ*g) from each sample was reverse-transcribed with oligo dT and random hexamer primers using reverse transcriptase (PrimeScript RT Enzyme Mix I, Takara RNA PCR Kit, Takara Biomedicals, Shiga, Japan). Real-time PCR was performed with 10 ng of cDNA and a pair of gene-specific primers (Takara Biomedicals) added to the SYBR Premix EX Taq (Takara Biomedicals) and subjected to PCR amplification in the iCycler iQ Real-Time Detection System (Bio-Rad Laboratories, Inc., Hercules, CA, USA). The expression of *β*-actin was used to normalize cDNA levels.

### 2.13. Statistical Analysis

Statistical differences between the various groups were assessed with a one-way analysis of variance (ANOVA) followed by post hoc Tukey's multiple-comparison test. Comparisons within groups were performed with a paired *t*-test. Neurological deficit scores were analyzed by the Kruskal-Wallis test, followed by the Mann-Whitney *U* test.

## 3. Results

### 3.1. Physiological Characteristic Parameters

KKAy mice showed moderate hyperglycemia and obesity typical in the type 2 diabetic model. Treatment with MAK (0.3 or 1 g/kg) for 1 week did not affect the body weight gain and blood glucose level of the mice (Table 2).

### 3.2. Effects of MAK on H/I-Induced Infarction and Neurological Deficits

**Figure 1:**
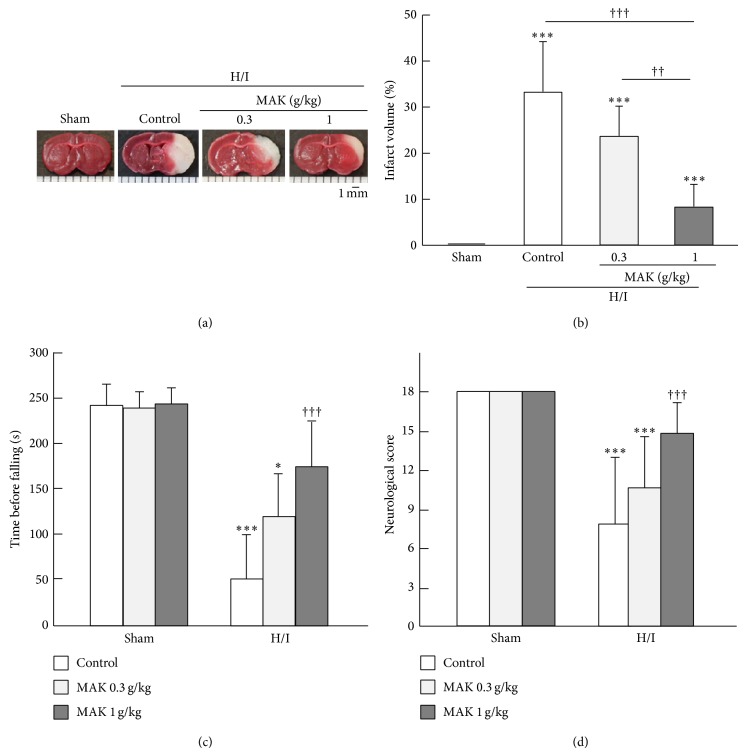
Effects of chronic pretreatment with MAK on H/I-induced infarction and neurological deficits. (a) Representative data of triphenyltetrazolium chloride (TTC) staining from the coronal brain sections at 24 h of reoxygenation after H/I in the mice. Scale bar = 1 mm. (b) The infarction volume was determined using an image analysis system. Throughout, the data are represented as means ± S.D.; *n* = 6–10 in each group. ^∗∗∗^ *P* < 0.001 compared with the sham-operated control group; ^††, †††^ *P* < 0.01, 0.001. (c) Motor coordination and balance at 24 h of reoxygenation after H/I were assessed by the rotarod performance test in the mice. ^∗, ∗∗∗^ *P* < 0.05, 0.001 compared with the respective sham-operated groups. ^†††^ *P* < 0.001 compared with the H/I-treated control group. (d) Neurological deficits were assessed using the 18-point neurological scoring system consisting of the neurological scoring index (Table 1) at 24 h of reoxygenation after H/I in the mice. The score for each mouse was summed for all five individual test scores. The maximum neurological score of a normal mouse with no deficit is 18. ^∗∗∗^ *P* < 0.001 compared with the respective sham-operated groups. ^†††^ *P* < 0.001 compared with the H/I-treated control group.

The infarction found in the TTC-stained coronal brain sections from the representative mice of each group at 24 h of reoxygenation after H/I is shown in Figure 1(a). The intact area of the tissue is stained with deep red originating from the mitochondrial activity of living cells, whereas the infarct area is pale pink. There was no apparent damage in the brain of sham-operated animals. In the control group, the infarction area was extended to the ipsilateral corpus striatum and the cortex during 24 h of reoxygenation after H/I. The corrected infract volume of the control mice was 33.1 ± 10.8% in KKAy, which was markedly increased compared with the value we obtained in the other experiment using nondiabetic C57BL/6J mice (1.8 ± 1.3%). The enlargement of cerebral infarction in KKAy was alleviated in the MAK groups and the values of infarct volume in 0.3 and 1 g/kg MAK groups decreased by 29.2% and 76.4% from that of the control group, respectively (Figure 1(b)). In the rotarod test, H/I and subsequent reoxygenation significantly impaired motor coordination and balance in the control group, whereas the sham-operation itself caused no deficit (Figure 1(c)). Pretreatment with MAK significantly and dose-dependently suppressed the deficits in locomotive function. In particular, the mice pretreated with 1 g/kg of MAK showed a markedly improved performance. In accordance with the results from the rotarod test, a severe neurological deficit in the control group was revealed by an 18-point neurological scoring system (Figure 1(d)). In contrast, the MAK (1 g/kg) group showed a better neurological score compared with the control group.

### 3.3. Effects of MAK on Neuronal Cell Death in the Ischemic Penumbra

**Figure 2:**
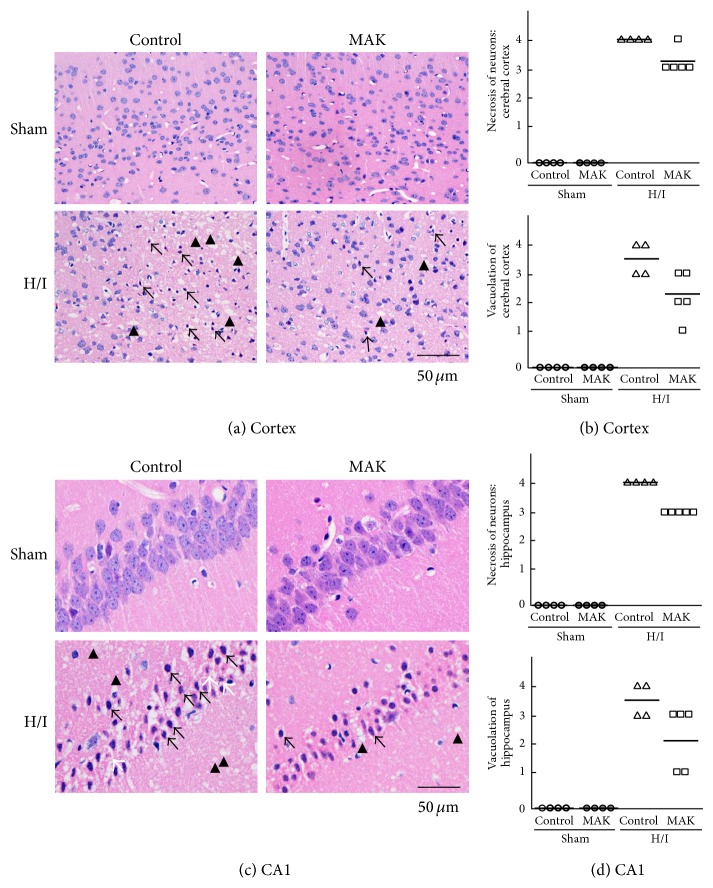
Effects of chronic pretreatment with MAK (1 g/kg) on neuronal cell death in the ischemic penumbra. Representative photographs obtained by H&E staining from the coronal brain sections, including the ischemic penumbral cortex (a) and hippocampus CA1 region (c) at 24 h of reoxygenation after H/I in the mice. Arrow: necrotic neuron with shrunken, often angular nucleus with hypereosinophilic cytoplasm, white arrow: apoptotic neuron with fragmented nucleus, and arrow head: vacuolation. The severity of the histopathological findings (upper panels: necrotic cell death, lower panels: vacuolation of the regions) in the ischemic penumbral cortex (b) and hippocampus CA1 region (d) was scored as (0) normal, (1) minimal, (2) mild, (3) moderate, and (4) marked; *n* = 4-5 in each group.

H&E staining of the ischemic penumbral region of the cortex (Figures 2(a) and 2(b)) and hippocampal CA1 region (Figures 2(c) and 2(d)) was performed to assess the H/I-induced tissue damage. In both the sham-operated control and MAK groups, H&E staining showed no abnormal neuronal cells in the cortex (Figure 2(a)) and hippocampus CA1 region (Figure 2(c)). On the other hand, marked cell atrophy and vacuolation of the parenchyma caused by H/I were observed in the ischemic cerebral cortex (Figure 2(a)). The nuclei of the neurons were visibly shrunken, often angular with hypereosinophilic cytoplasm (arrow). As shown in Figure 2(c), H/I caused serious pathological alterations in the ischemic hippocampal CA1 region. Nuclei of many remaining neurons were shrunken with eosinophilic cytoplasm. They exhibited the hallmark features of necrotic neurons (arrow). Several atrophic neurons produced nuclear fragments, conceivable apoptotic bodies (white arrow). Abundant vacuolation (arrow head), indicating degeneration and collapse of nerve fiber projections of neurons, emerged in the hippocampus. MAK (1 g/kg) was able to ameliorate the abovementioned pathological changes both in cerebral cortex and hippocampus (Figures 2(b) and 2(d)).

### 3.4. Effects of MAK on Oxidative Stress in Plasma and Brain

**Figure 3:**
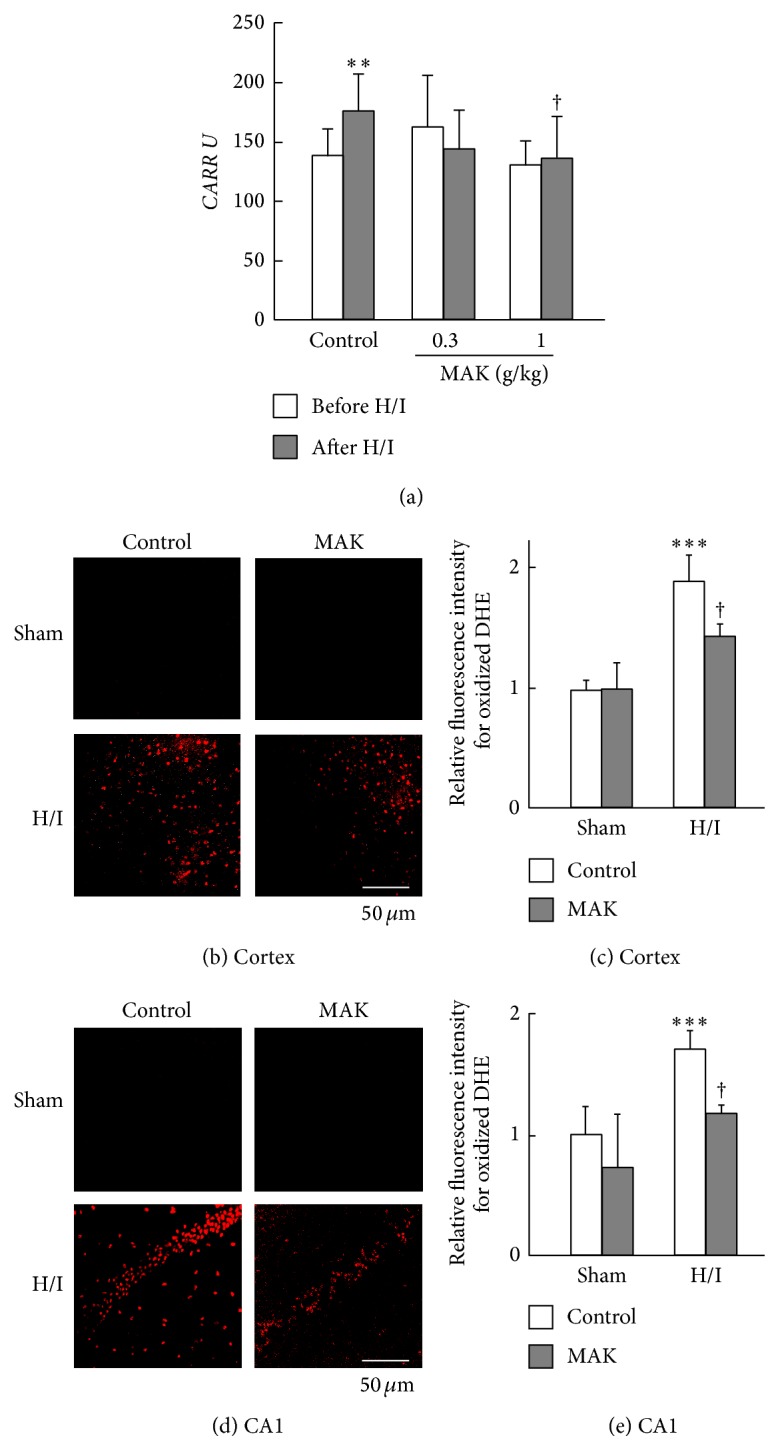
Effects of chronic pretreatment with MAK on systemic and cerebral oxidative stress after H/I. (a) The levels of total plasma oxidative stress before H/I and at 24 h of reoxygenation in each group were determined by the d-ROMs test.; *n* = 6–10 in each group. ^∗∗^ *P* < 0.01 compared with the control group before H/I; ^†^ *P* < 0.05 compared with the control group after H/I. Representative data of DHE staining for superoxide production at 24 h of reoxygenation after H/I in the penumbral cortex (b) and hippocampal CA1 region (d) from the mice in each group. MAK was administered at a dose of 1 g/kg. Scale bar = 50 *μ*m. Fluorescence intensity of the oxidized DHE was quantified using imaging software in the penumbral cortex (c) and hippocampal CA1 region (e). The values of fluorescence intensity of each group are represented as means ± S.D. relative to those of the cortex in the control group; *n* = 3–5. ^∗∗∗^ *P* < 0.001, compared with the sham-operated control group; ^†^ *P* < 0.05 compared with the H/I-treated control group.

To assess the effects of MAK on H/I-induced systemic oxidative stress, plasma levels of hydroperoxide in the control and MAK groups were measured by the d-ROMs test at 24 h of reoxygenation after H/I (Figure 3(a)). H/I and reoxygenation significantly increased this parameter in the control group, which was absent in the MAK group.

Intracellular O_2_^−^ generation induced by H/I in the penumbral cortex (Figures 3(b) and 3(c)) and hippocampal CA1 region (Figures 3(d) and 3(e)) was detected by histological staining with a fluorescent probe DHE. At 24 h of reoxygenation after H/I, O_2_^−^ generation was remarkably augmented in neuronal cells in the control brain and significantly suppressed in the MAK (1 g/kg)-treated brain. There was no difference in the amount of O_2_^−^ generation of the regions between control and MAK groups with sham operation.

### 3.5. Effects of MAK on Neuronal Apoptosis

**Figure 4:**
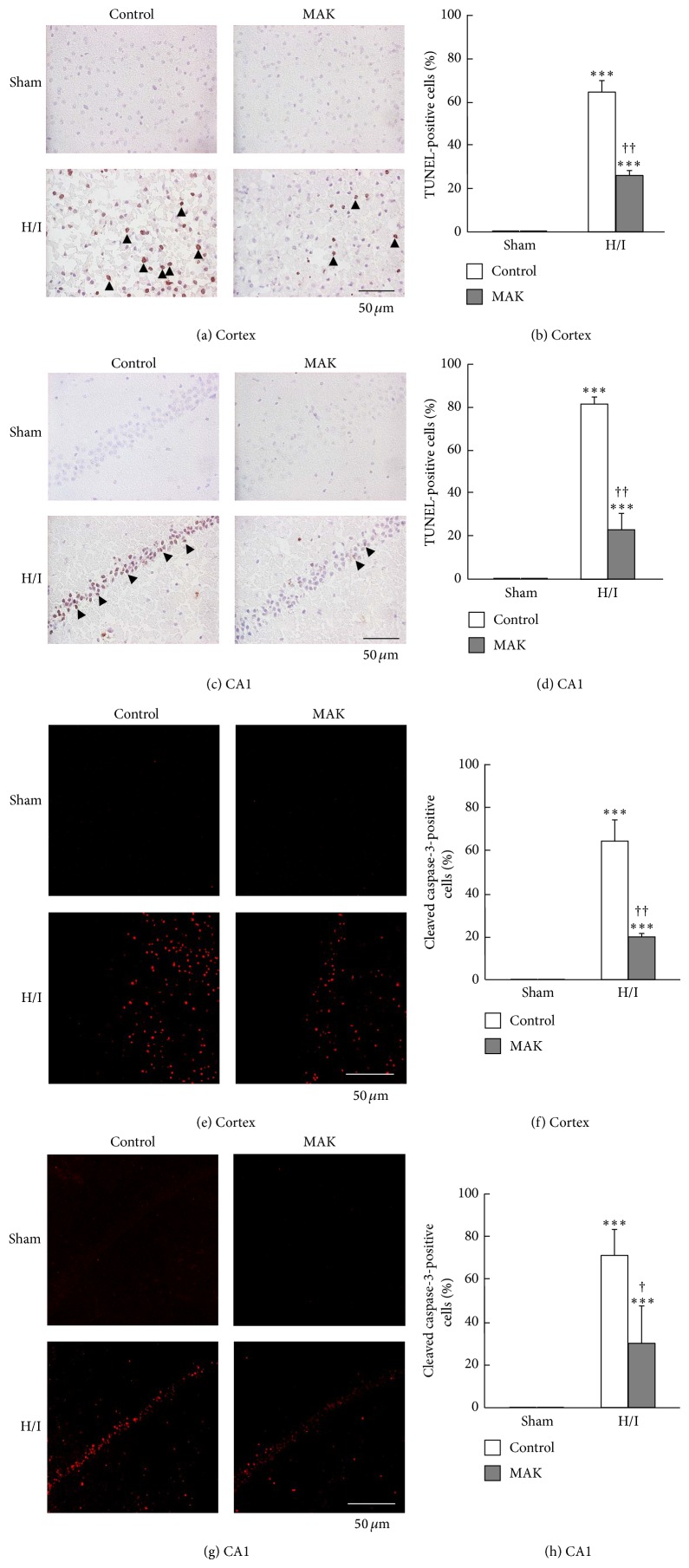
Effects of chronic pretreatment with MAK (1 g/kg) on apoptotic cell death in the ischemic penumbral regions. Representative photographs of TUNEL staining and cleaved caspase-3 immunostaining at 24 h of reoxygenation after H/I in the penumbral cortex (a), (e) and hippocampal CA1 region (c), (g) from the mice in each group. Scale bar = 50 *μ*m. Quantification of the number of TUNEL-positive (arrow heads) or cleaved caspase-3-positive cells was achieved by cell counting in the penumbral cortex (b), (f) and hippocampal CA1 region (d), (h). Throughout, data are represented as means ± S.D. *n* = 3–5. ^∗∗∗^ *P* < 0.001 compared with the respective sham-operated groups. ^†, ††^ *P* < 0.05, 0.01 compared with the H/I-treated control group.

TUNEL staining of the ischemic penumbral region of the cortex (Figures 4(a) and 4(b)) and hippocampal CA1 region (Figures 4(c) and 4(d)) was performed to determine nucleosomal DNA fragmentation accompanied by apoptotic cell death. In both the sham-operated control and the MAK groups, no TUNEL-positive cells were detected in the regions. The number of TUNEL-positive cells was increased in the control group by H/I and subsequent reoxygenation and remarkably suppressed by pretreatment with MAK. The activation level of caspase-3 which directly activates DNase in the apoptotic final process was determined by immunostaining for cleaved caspase-3, an activated form of this enzyme. Similar to the result of TUNEL staining, the number of cells expressing cleaved caspase-3 was remarkably increased by H/I and subsequent reoxygenation in the control group (Figures 4(e)–4(h)). Pretreatment with MAK significantly inhibited the caspase-3 activation induced by H/I and subsequent reoxygenation.

### 3.6. Effects of MAK on Gene Expression of Bcl-2 and Bax

**Figure 5:**
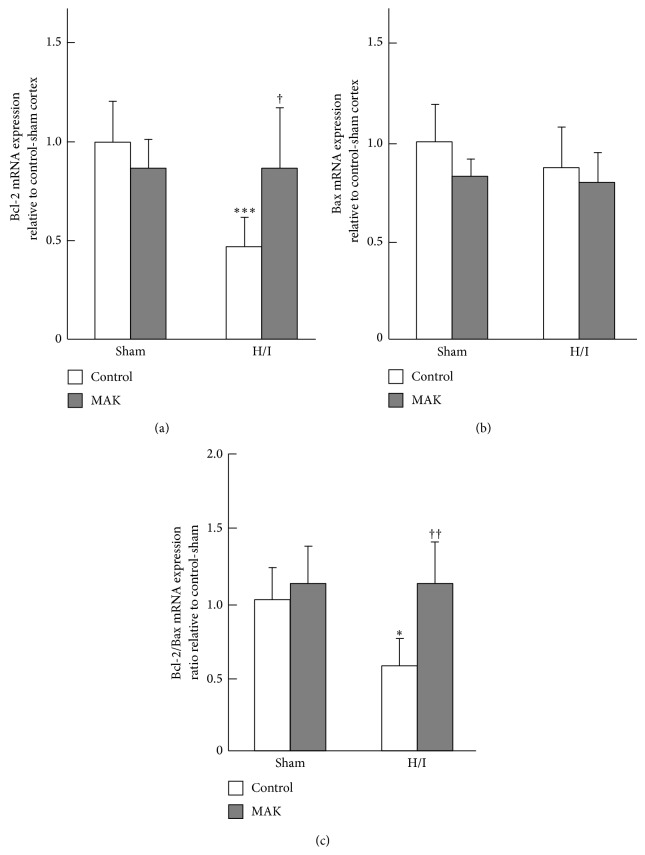
Effects of chronic pretreatment with MAK (1 g/kg) on expression of apoptosis factors gene expression after H/I. Expression levels of Bcl-2 (a) and Bax (b) mRNA after H/I at 24 h of reoxygenation after H/I in the penumbral cortex from the mice in each group, determined by real-time RT-PCR analysis. (c) The Bcl-2/Bax ratio was calculated. *n* = 6-7 in each group. ^∗, ∗∗∗^ *P* < 0.05, 0.001 compared with the sham-operated control group, ^†, ††^ *P* < 0.05, 0.01 compared with the H/I-treated control group.

The mRNA expression levels of the apoptosis-related genes encoding Bcl-2 (Figure 5(a)) and Bax (Figure 5(b)) in the penumbral cortex were quantified by real-time PCR. H/I and subsequent reoxygenation significantly decreased the expression level of Bcl-2 mRNA in the control group, whereas it did not change the level of Bcl-2 mRNA in the MAK (1 g/kg) group. On the other hand, the expression levels of Bax mRNA were not significantly different between groups. Therefore, the ratio of Bcl-2/Bax mRNA, which reflects a change in the balance between antiapoptosis and apoptosis-promoting genes, was significantly decreased in the control group compared with the sham-operated control group, whereas this ratio in the MAK group was almost equal to that of the sham-operated MAK group (Figure 5(c)).

### 3.7. Effects of MAK on Gene Expression of RIP1 and RIP3

**Figure 6:**
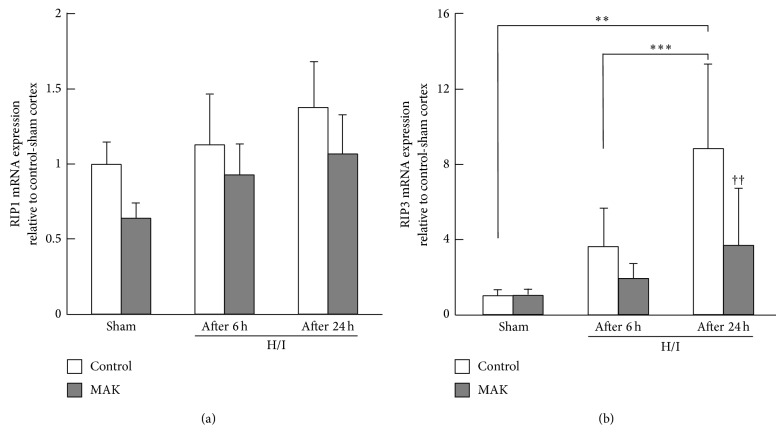
Effects of chronic pretreatment with MAK (1 g/kg) on the expression of necroptotic factor gene expression after H/I. Expression levels of RIP1 (a) and RIP3 (b) mRNA at 6 (after 6 h) or 24 h (after 24 h) of reoxygenation after H/I in the penumbral cortex from the mice in each group, determined by real-time RT-PCR analysis.; *n* = 6–10 in each group. ^∗∗, ∗∗∗^ *P* < 0.01, 0.001. ^††^ *P* < 0.01 compared with the H/I-treated control group.

The expression levels of RIP1 and RIP3 mRNA in the penumbral cortex for each group were quantified by real-time PCR. The expression of RIP1 mRNA showed no significant change by either H/I or MAK (1 g/kg)-treatment (Figure 6(a)). On the other hand, H/I increased the expression level of RIP3 mRNA, depending on the duration of reoxygenation (Figure 6(b)). RIP3 mRNA upregulation was prevented by pretreatment with MAK.

### 3.8. Effects of MAK on Expression of RIP3 in the Ischemic Penumbra

**Figure 7:**
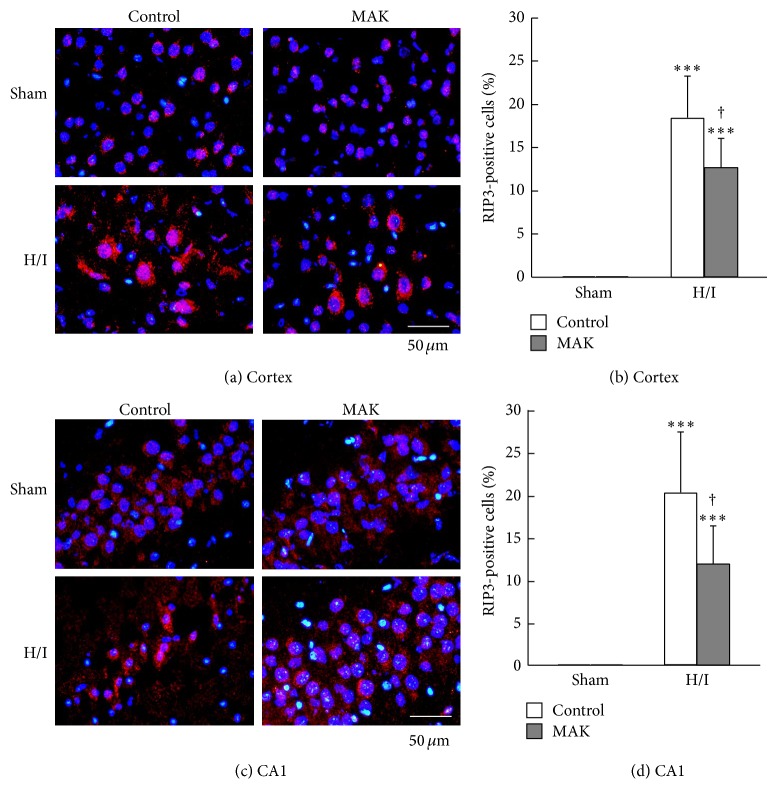
Effects of chronic pretreatment with MAK (1 g/kg) on RIP3 expression in the ischemic penumbral regions. (a) Representative photographs of RIP3 immunostaining at 24 h of reoxygenation after H/I in the penumbral cortex (a) and hippocampal CA1 region (c) from the mice in each group. Scale bar = 50 *μ*m. Quantification of the number of RIP3-positive cells was achieved by cell counting in the penumbral cortex (b) and hippocampal CA1 region (d) from the mice in each group. Throughout, data are represented as means ± S.D. from 3–5 mice in each group. ^∗∗∗^ *P* < 0.001 compared with the respective sham-operated groups. ^†^ *P* < 0.05 compared with the H/I-treated control group.

Figure 7 shows representative photographs of RIP3 immunostaining of the penumbral cortex and hippocampal CA1 region from each group. A lower level of the expression of RIP3 was observed in the cortex of control and MAK sham-operated groups. At 24 h of reoxygenation after H/I, the number of RIP3-positive cells that showed a markedly enhanced expression of RIP3 protein was increased both in the cortex (Figures 7(a) and 7(b)) and CA1 (Figures 7(c) and 7(d)), which was significantly suppressed by pretreatment with MAK.

### 3.9. Effects of MAK on Gene Expression of TNF-*α*

**Figure 8:**
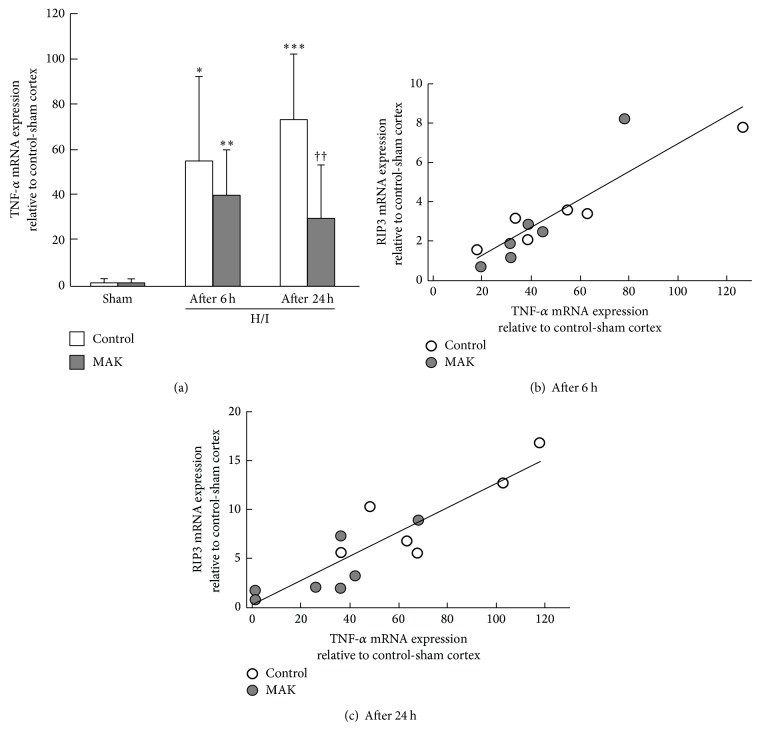
Effects of chronic pretreatment with MAK (1 g/kg) on expression of TNF-*α* gene expression after H/I. (a) Expression levels of TNF-*α* mRNA at 6 (after 6 h) or 24 h (after 24 h) of reoxygenation after H/I in the penumbral cortex from the mice in each group, determined by real-time RT-PCR analysis; *n* = 6-7 in each group. ^∗, ∗∗, ∗∗∗^ *P* < 0.05, 0.01, 0.001 compared with the respective sham-operated groups. ^††^ *P* < 0.01 compared with the H/I-treated control group. The relative expression values of TNF-*α* mRNA were plotted versus those of RIP3 mRNA obtained from all individual penumbral cortices at 6 (b) and 24 h (c) of reoxygenation after H/I. The data were pooled from the control (open circles) and MAK-pretreated (closed circles) mice.

Because the process of necroptosis can be triggered by the TNF superfamily, we measured the expression levels of TNF-*α* mRNA in the penumbral cortex from each group (Figure 8(a)). H/I markedly upregulated the gene expression of TNF-*α* in the control brain, which was significantly suppressed by the pretreatment with MAK. The relative expression values of TNF-*α* mRNA were plotted versus those of RIP3 mRNA obtained from all individual penumbral cortices at 6 (Figure 8(b)) and 24 h (Figure 8(c)) of reoxygenation, indicating a positive correlation between the expression values of TNF-*α* mRNA and those of RIP3 mRNA (correlation coefficient: 0.79 and 0.80 for the data from 6 and 24 h after reoxygenation, resp.).

## 4. Discussion

In the present study, we demonstrated that 1-week oral pretreatment with MAK exerts moderate but significant protective effects against H/I-induced brain infarction and neurological deficits in type 2 diabetic KKAy mice. MAK decreased the numbers of TUNEL- or cleaved caspase-3-positive cells in the penumbral cortex and hippocampus, suggesting that MAK can protect neuronal cells against H/I-induced apoptosis in the mice. In addition, MAK markedly decreased necrotic cell atrophy and vacuolation, which was associated with a downregulated expression of RIP3, a key molecule of necroptosis, in the ischemic penumbra. These results suggest that chronic pretreatment with MAK exerts significant antiapoptotic and antinecroptotic effects in the ischemic brain of the type 2 diabetic mice.

MAK has been used as a revitalizer for 18 years, and a number of studies have demonstrated its antitumor [42] and immunomodulating activities [43]. However, the mechanistic basis and active ingredients responsible for its pharmacological effects have not been well defined. MAK is composed of bagasse and defatted rice bran that is overgrown with* G. lucidum* mycelia, which are considered to contain various bioactive substances, including triterpenes, polysaccharides, water-soluble lignin, and its degradation products such as syringic acid and vanillic acid. Our group has revealed that MAK exerts antioxidative activities* in vitro *[44] and* in vivo* [32–34, 44]. MAK shows antidiabetic effects by relieving oxidative stress in STZ-induced type 1 diabetic animals; MAK ameliorates lipid peroxidation and dysfunction of antioxidative enzymes (superoxide dismutase, catalase, and glutathione peroxidase) in the brain [32], liver, and kidney [44] in diabetic animals. Furthermore, MAK prevents MCAO/reperfusion-induced apoptosis and inflammatory responses in neuronal cells and reduces the size of cerebral infarction in STZ-treated rats [32, 33]. The safety of MAK has been confirmed by* in vitro* toxicological evaluation and animal toxicity studies, and there has been no report suggesting that MAK had potential acute or long-term toxicities in consumers in the past, supporting the assertion that 1-week treatment with 1 g/kg MAK used in the present study has little or no toxicity.

Previous basic studies have demonstrated that both type 1 [6–8] and type 2 diabetes [9, 10] augment oxidative stress and aggravate ischemic injury in the brain. The augmentation of the intracellular glucose oxidation process and nonenzymatic glycation of protein molecules enhanced by diabetic hyperglycemia is considered to lead to the overproduction of ROS and oxidative damage of neurons, triggering the apoptotic process in the cells [7, 11, 45]. Apoptotic cell death in the ischemic penumbra has been demonstrably enhanced by diabetes and correlated with serum glucose [6]. Our current study showed that oral pretreatment with MAK suppressed H/I-induced enhancement of hydroperoxide production in the plasma, O_2_^−^ generation, and apoptotic cell death in the ischemic penumbra of KKAy mice, which could be attributed to the improved antioxidant status and reduced oxidative stress in the diabetic state. We observed that MAK decreased H/I-induced neuronal cell death in KKAy mice without any significant effects on body weight gain or blood glucose, confirming that the cerebroprotective effects of MAK could be due to its antioxidant activity.

Mitochondria play an essential role in cerebral ischemia with diabetes [46]. Hyperglycemia has been suggested to impair mitochondrial functions and induce excess ROS production, leading neuronal cells to apoptosis. Mitochondrial-formed oxidants are mediators of molecular signaling in the mitochondria-dependent intrinsic apoptosis pathway involving cytochrome *c* release from the mitochondria into the cytosol as a crucial process [47]. In this pathway, a proapoptotic factor Bax protein increases the permeability of the mitochondrial membrane by translocation from the cytoplasm to the membrane and initiates cytochrome *c* release; Bcl-2 is an antiapoptotic protein which counteracts Bax, binding to the outer mitochondrial membrane, maintaining membrane integrity, and enhancing cell survival [48, 49]. Therefore, the Bcl-2/Bax ratio can be taken as an index responsible for the execution of mitochondria-dependent apoptosis. In our study, the Bcl-2/Bax mRNA ratio was decreased by H/I, suggesting that H/I caused mitochondrial dysfunction by mitochondrial-generated ROS accumulation in the cells. Pretreatment with MAK increased the levels of antiapoptotic Bcl-2 mRNA and the Bcl-2/Bax mRNA ratio in the ischemic penumbra. MAK also markedly decreased the number of cells overexpressing activated caspase-3, the executioner of apoptosis in the ischemic penumbral regions [48]. In addition, we showed that MAK prevented the H/I-induced increase in TNF-*α* mRNA expression in the penumbral cortex. The extrinsic pathway of apoptosis has been clarified to be triggered by the activation of the TNF superfamily cell-death receptors, which recruits other proteins to form a complex that ultimately activates caspase-3 [50]. Overall, our present results suggest that MAK inhibits apoptotic cell death in the ischemic penumbra by concomitant inhibition of intrinsic and extrinsic pathways of apoptosis, presumably via an increase in the Bcl-2/Bax ratio and suppression of the expression of TNF-*α*.

In the exacerbation mechanisms of poststroke brain damage with diabetes, a magnified inflammation and associated uncontrolled necrotic cell death are critically involved [15]. H&E staining revealed that MAK markedly decreased necrotic cell death induced by H/I and reoxygenation in the penumbral cortex and hippocampus. Necrotic cell death facilitates inflammatory responses during the acute period after ischemia, because necrotic cells rapidly lose plasma membrane, which leads to the release of immunostimulatory adjuvants such as high-mobility group box protein 1 from the cells, triggering inflammation storm [51]. This necrosis was considered to occur incidentally and to be uncontrollable, until necroptosis, a novel type of programmed and controllable necrosis extrinsically triggered by the TNF superfamily, became recognized [19]. For the execution of necroptosis, the kinase activity of RIP3 is required [26, 27]. Several groups have demonstrated that the ischemic condition upregulates the expression of RIP3 in neurons [21, 28]. Vieira et al. [28] have shown that RIP3 knockdown suppresses the oxygen-glucose deprivation-induced necroptosis whereas RIP3 overexpression enhances the cell death in hippocampal culture neurons, suggesting an involvement of regulation of the expression of RIP3 in necroptosis pathway. Therefore, we investigated the effects of MAK on the expression of RIP3 in the ischemic penumbral regions of KKAy mice. Immunohistochemistry revealed that H/I and subsequent reoxygenation upregulated the expression of RIP3 in both the cortex and hippocampus. This was compatible with the data showing an upregulation in gene expression of RIP3. We observed that pretreatment with MAK prevented the H/I-induced RIP3 mRNA and protein upregulation in the ischemic penumbra. Furthermore, we found that the gene expression levels of TNF-*α* and RIP3 were positively correlated, suggesting that TNF-*α* could positively regulate RIP3 transcription. MAK may alleviate the H/I-induced RIP3 upregulation via a TNF-*α* downregulation in the neuronal cells of the ischemic penumbra. Accumulated adipose tissue-induced dysregulated production of adipocytokines occurring in visceral obesity associated with type 2 diabetes has been reported to induce the production of proinflammatory cytokines, including TNF-*α*, and augment oxidative stress [12, 13]. Previously, we indicated that pretreatment with MAK suppresses focal cerebral ischemia-induced activation of nuclear factor (NF)-*κ*B, a transcription factor responsible for inflammation leading to abundant TNF production in microglia, and decreased expression of TNF-*α*, IL-1*β*, COX-2, and MPO in the cells of the type 1 diabetic rat brain [33]. Recently, a study has shown that* G. lucidum* fungus mixture obtained by nearly the same preparation method as that of MAK suppresses the expression of TNF-*α* in the hippocampus after MCAO/reperfusion in rats [52], which is consistent with our findings. Other studies have reported that polysaccharides [53] and triterpenes [54] extracted from the* G. lucidum *fruit body suppress lipopolysaccharide-induced expression of inflammatory mediators via an inhibition of NF-*κ*B activity. Therefore, one possibility is that MAK inhibits inflammatory responses via inhibition of monocyte activation. Further experiments are needed to elucidate more detailed neuroprotective mechanisms of MAK and to identify its active ingredients.

## 5. Conclusions

In conclusion, chronic treatment with MAK prevents necroptotic cell death as well as apoptosis induced by H/I in KKAy mice brain, which may be attributed to its antioxidant and anti-inflammatory activities. MAK downregulates TNF-*α* in the ischemic penumbra, which may abrogate RIP3 upregulation and prevent the neuronal necroptosis.

## Figures and Tables

**Table 1:** Neurological scoring index [30].

	Scores			
	0	1	2	3
Spontaneous activity, 3 min	No movement	Slight movement	Touches 1-2 sides of the cage	Touches 3-4 sides of the cage

Symmetry of movement, right forelimb, and hind limb	Total asymmetry	Near-total asymmetry	Mild asymmetry	Complete symmetry

Floor walking	No walking	Walks only in circles	Curvilinear path	Straight path

Beam walking	Falls off the beam	Hugs the beam	Stands on the beam	Walks on the beam

Response to vibrissae touch		No response	Weak response	Symmetrical response as compared to unaffected side

Side stroking		No response	Weak response	Symmetrical response as compared to unaffected side

**Table 2:** Body weight, feed intake, and blood glucose in mice during MAK treatment.

Group	Body weight (g)		Feed intake (g)		Blood glucose (mg/dL)	
	Day 0	Day 7	Day 0	Day 7	Day 0	Day 7
Control (H_2_O)	37.2 ± 1.3	38.4 ± 1.5	6.0 ± 0.8	6.3 ± 0.6	350 ± 119	383 ± 103
MAK 0.3 g/kg	37.6 ± 1.3	37.5 ± 1.9	6.1 ± 0.6	6.3 ± 0.6	375 ± 193	358 ± 133
MAK 1 g/kg	37.1 ± 1.1	37.7 ± 1.1	6.3 ± 0.6	6.2 ± 0.6	352 ± 116	346 ± 97

Results are the mean ± S.E.M. The number of mice per group was, in control group, *n* = 10; in MAK- (0.3 g/kg) treated group, *n* = 6; in MAK- (1 g/kg) treated group, *n* = 9.
